# Joseph T. Freeman Award Lecture

**DOI:** 10.1093/geroni/igaa057.3206

**Published:** 2020-12-16

## Abstract

The Joseph T. Freeman Award lecture will feature an address by the2020 Freeman Award recipient Cynthia Brown, MD, MSPH. The Joseph T. Freeman Award is a lectureship in geriatrics awarded to a prominent physician in the field of aging, both in research and practice. The award was established in1977 through a bequest from a patient’s estate as a tribute to Dr. Joseph T. Freeman.

